# Aggregation-Induced Generation of Reactive Oxygen Species: Mechanism and Photosensitizer Construction

**DOI:** 10.3390/molecules26020268

**Published:** 2021-01-07

**Authors:** Juechen Ni, Yijia Wang, Haoke Zhang, Jing Zhi Sun, Ben Zhong Tang

**Affiliations:** 1 MOE Key Laboratory of Macromolecular Synthesis and Functionalization, Department of Polymer Science and Engineering, Zhejiang University, Hangzhou 310027, China; 11929043@zju.edu.cn (J.N.); zhanghaoke@zju.edu.cn (H.Z.); 2 Key Laboratory of Advanced Textile Materials and Manufacturing Technology, Ministry of Education, College of Textiles, Zhejiang Sci-Tech University, Hangzhou 310018, China; wangyijia@zstu.edu.cn; 3 Department of Chemistry, The Hong Kong Branch of Chinese National Engineering Research Center for Tissue Restoration and Reconstruction and Institute for Advanced Study, The Hong Kong University of Science and Technology, Clear Water Bay, Kowloon, Hong Kong 999077, China; 4 Center for Aggregation-Induced Emission, SCUT-HKUST Joint Research Institute, State Key Laboratory of Luminescent Materials and Devices, South China University of Technology, Guangzhou 510640, China

**Keywords:** aggregation-induced emission, reactive oxygen species, electron transfer, energy transfer, photodynamic therapy

## Abstract

Luminogens with aggregation-induced emission (AIEgens) have been widely applied in the field of photodynamic therapy. Among them, aggregation-induced emission photosensitizers (AIE–PSs) are demonstrated with high capability in fluorescence and photoacoustic bimodal imaging, as well as in fluorescence imaging-guided photodynamic therapy. They not only improve diagnosis accuracy but also provide an efficient theranostic platform to accelerate preclinical translation as well. In this short review, we divide AIE–PSs into three categories. Through the analysis of such classification and construction methods, it will be helpful for scientists to further develop various types of AIE–PSs with superior performance.

## 1. Introduction

Although important advances in cancer diagnosis and therapy have been achieved over recent decades, cancer patients are still suffering from severe side effects and constant relapses [1,2]. Fortunately, photodynamic therapy (PDT) has been investigated as a noninvasive modality for cancer treatment owing to its high selectivity and low side effects since the end of 20th century [3,4]. A PDT system is usually composed of three separately nontoxic parts: a photosensitizing drug or photosensitizer (PS), a light source that emits visible and/or near-infrared (NIR) photons and molecular oxygen dissolved in the target tissue [5], and among them, PSs can transfer photon energy to surrounding oxygen molecules to produce reactive oxygen species (ROS); in particular, singlet oxygen (^1^O_2_) that causes cell death [6] is the most important one.

**Figure 1:**
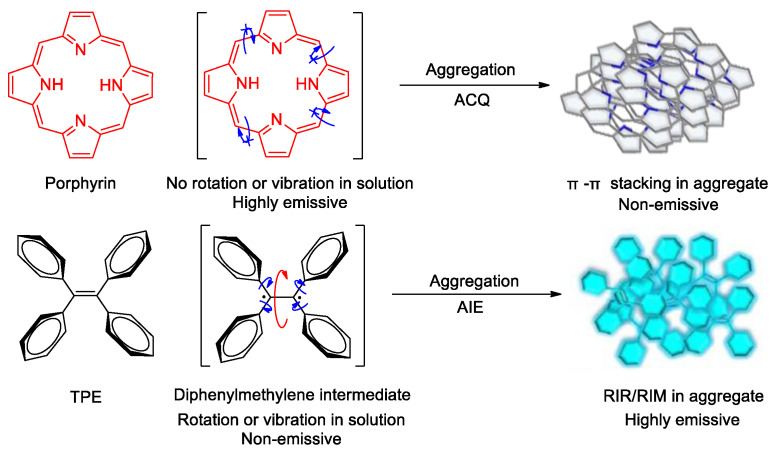
Schematic diagrams illustrating fluorescence quenching of traditional photosensitizers (PSs) such as porphyrin in their aggregate states vs. fluorescence turn-on effect of aggregation-induced emission (AIE) fluorogens such as tetraphenylethene (TPE) upon aggregate formation. Adapted and modified with permission from reference 15 (copyright 2015, American Chemical Society).

Porphyrins and phthalocyanines are the most widely studied PSs with fast intersystem crossing and long triplet lifetime and have already been applied in clinical applications [7,8]. Nevertheless, these PSs usually aggregate in aqueous media due to their low water solubility. This leads to low fluorescent intensity and PDT efficiency owing to the aggregation-caused quenching (ACQ) effect (Figure 1), which makes image-guided PDT difficult to achieve [9,10,11]. To solve this problem, some water-soluble and nonaggregating PSs have been synthesized [12]. However, such an approach greatly increased the cost of material preparation [13]. So, it is of great importance and is meaningful to develop novel PSs with outstanding fluorescent property in aqueous environments.

In 2001, a new concept termed as aggregation-induced emission (AIE) was proposed by Tang and co-workers [14,15], and since then, a series of luminogens with aggregation-induced emission property (AIEgens) have been developed. A typical AIEgen shows neglectable emission in dilute solutions but much enhanced emission in molecular ensembles [16,17]. In other words, AlEgens can utilize aggregation to play a positive instead of a negative role in enhancing luminescence [18]. The systematic study of the mechanism has profoundly revealed the molecular mechanism of the AIE phenomenon: molecules with AIE characteristics often have a propeller-like configuration—a number of conjugated units are connected through single bonds that can rotate [19]. In dilute solutions, the conjugated units within the molecule can rotate upon the promotion of solvent molecules. When molecules are excited by light or other energy sources, these rotations consume almost all of the excited state energy (non-radiation decay, e.g., thermal motion), and the molecules emit very weak luminescence or are even non-emissive. When the molecules aggregated, the conjugated units can be bound by the enhanced intermolecular interactions, and their rotations are evidently inhibited. Thus, the channels for non-radiation decay are largely closed and the excited state energy of the molecules is released in the form of fluorescence/phosphorescence (radiation decay). As a result, luminescence can be observed. This mechanism for AIE phenomenon is termed as restricted intramolecular rotation (RIR) [19,20,21,22]. On the other hand, AIE properties have been observed for some conjugated molecules with shell or butterfly wing-like structures [23,24]. The results of systematic study indicate that their AIE behavior is due to the molecular vibration. The opening/closing of the shells or the flapping of the butterfly wings of these kinds of molecules consume their excited state energy, causing them with lack emission in dilute solution, while they are strongly emissive in the aggregated state as the intramolecular vibrations are restricted. This is another mechanism of AIE phenomenon explained as restricted intramolecular vibration (RIV) [25]. Nowadays, the RIR and RIV mechanisms are integrated into the restricted intramolecular motions (RIM) theory for AIE phenomenon [15].

AIEgens with free rotational/vibrational structures can enhance the intersystem crossing (ISC) between the excited singlet state (S_1_) and the excited triplet state (T_1_), and thereby increase the quantum yield and lifetime of the triplet state, as well as the efficiency of ROS generation. Taking advantage of the high-efficiency luminescence in an aggregated state, nanoparticles prepared from AIE molecules (AIE–dots) can exhibit strong luminescence as well as good photostability in the bio-system, and effectively overcome the shortcomings of the traditional dye with ACQ property. Therefore, aggregation-induced emission photosensitizers (AIE–PSs) can present huge potential in the field of imaging-guided PDT [26,27].

In this short review, we will outline the latest developments in AIE–PSs from the perspective of the mechanism by which PSs produce ROS. We will first introduce the mechanism of aggregation-induced generation reactive oxygen species. The AIE–PSs will be classified into Type I and Type II according to the production methods of the reactive oxygen species, which are electron transfer and energy transfer between PSs and oxygen, respectively. Moreover, some AIE–PSs that produce ROS in two pathways: Type I and Type II, or produce both singlet oxygen (^1^O_2_) and reactive oxygen radicals, will also be introduced. Finally, we will clarify the limitations, challenges and future opportunities of ROS generation based on AIE–PSs.

## 2. Aggregation-Induced ROS Generation Mechanism

**Figure 2:**
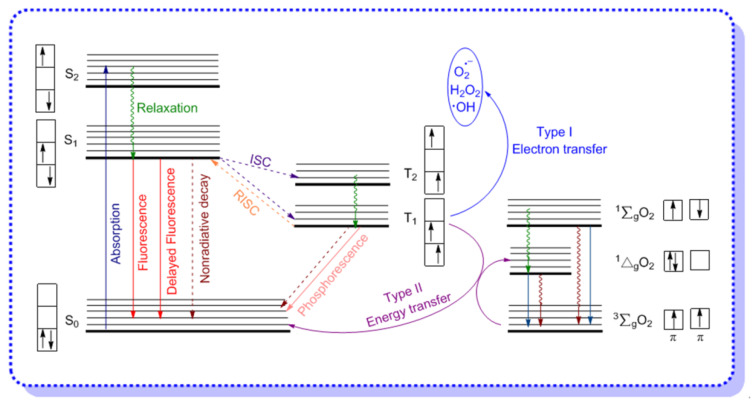
Photophysical and photochemical processes illustrated by a modified Jablonski energy diagram: The aggregated aggregation-induced emission photosensitizer (AIE–PS) in singlet excited (S_1_) state may undergo intersystem crossing (ISC) to an excited triplet state (T_1_) and then generate reactive oxygen species (ROS). Adapted and modified with permission from references 5 (copyright 2017, Elsevier), 6 (copyright 2016, Royal Society of Chemistry), and 16 (copyright 2020, Wiley-VCH).

Absorption of a photon leads to the formation of an “excited state” of the photosensitizing agent. As illustrated in Figure 2, the common features of the electronic excitation and the consequent photochemical reactions may be described by three electronic states: singlet ground state (S_0_), singlet excited states (S_1_, S_2_), and longer-lived triplet excited state (T_1_).

Electronic absorption occurs between the vibrational and rotational energy levels of the excited singlet states. Immediately after photon absorption, several photophysical and photochemical processes occur, but the most likely deactivation pathway is a relaxation to the lowest vibrational energy level of the first excited state. This very fast process (10^−15^ s) is defined as relaxation. For AIE–PSs in the aggregated state, the non-radiative decay process such as intramolecular rotation (RIR) and intramolecular vibration (RIV) will be restricted in the excited state. If relaxation from S_1_ is accompanied by emission of a photon, the process is known as fluorescence. Several other relaxation pathways compete with the photonic processes are also described above, and among them, the most important one is a nonradiative process to the lowest excited triplet state (T_1_), which is known as intersystem crossing (ISC). The latter event may result in either the emission of a photon from triplet excited state (T_1_) through spin-forbidden phosphorescence, or the photochemical reactions. It is noteworthy that molecules in the excited states are generally stronger oxidizing and reducing species than their analogues in the ground states. Thus, apart from the radiative decay processes of fluorescence and phosphorescence, the exciton may also undergo internal conversion to release energy as heat, or generate ROS by the electron transfer (Type I)/energy transfer (Type II) of triplet oxygen [28,29].

Before introducing the mechanism of electron transfer and energy transfer while generating ROS, we need to understand the electronic structure of singlet oxygen and triplet oxygen first. As shown in Figure 2, molecular oxygen has two low-lying singlet excited states, the first excited state (^1^Δ_g_) and the second excited state (^1^∑_g_), which are 95 and 158 kJ mol^−1^ above the triplet state (^3^∑_g_), respectively, and the only difference in their electronic configuration is the structure of the π-antibonding orbitals. For ^1^Δ_g_, the configuration of the molecular orbitals is: O_2_ [KK(σ_2s_)^2^(σ*_2s_)^2^(σ_2px_)^2^(π_2p_)^4^(π*_2py_)^2^(π*_2pz_)^0^], while for ^1^∑_g_, the electronic configuration is identical to that of the ground state, except that the last two electrons have antiparallel spins. Normally, the transition from the ^1^Δ_g_ state to the ^3^∑_g_ state is spin forbidden, which means that the ^1^Δ_g_O_2_ is a relatively long-lived species, meanwhile, the second excited state of oxygen is short-lived due to its spin-allowed transition to the ^1^Δ_g_ state, and all of these have been confirmed by the radiative lifetimes of O_2_ (^1^Δ_g_) and O_2_ (^3^∑_g_) [30].

### 2.1. Electron Transfer (Type I) Mechanism

As reported in the literature, molecules in the excited state always have larger oxidation/reduction potential than their ground state analogs [5]. AIE–PSs can generate superoxide anion (O_2_^•−^), hydroxyl radical (•OH), peroxide (O_2_^2−^), and other free radical ROS through electron transfer under low concentrated oxygen conditions. The first oxygen-centered radical generated according to Type I mechanism is O_2_^•−^. It is formed when an electron is captured by one of the π*_2p_ orbitals of oxygen either in the presence of a reducing agent or directly from an excited photosensitizer.

The reduction potential of superoxide ion may change with the surrounding environment. For example, O_2_^•−^ is a weak oxidizing agent in aqueous media, but it is still able to oxidize to, for example, ascorbic acid [31]. Meanwhile, it can also act as a strong reducing agent and enable the reduction of iron complexes in cytochrome *c*, as well as in the ferric/ethylene diamine tetraacetic acid (EDTA) complex [32]. The subsequent perhydroxyl radical is formed. The perhydroxyl radical is a more potent oxidant than superoxide ion, thus it is able to oxidize O_2_^•−^ and resulting in the H_2_O_2_ formation. Dismutation of O_2_^•−^ is often occur under the catalysis of superoxide dismutase (SOD) or PS, and leads to the formation of hydrogen peroxide (H_2_O_2_), which is characterized by a much longer half-life than other kinds of ROS. In contrast with other kinds of ROS, H_2_O_2_ can pass through biological membranes and cause the damage of other cellular compartments [33]. Moreover, H_2_O_2_ can be detoxified by the enzyme catalase, resulting in the formation of water and molecular oxygen, or may further react with either superoxide ion, or form highly reactive hydroxyl radicals. The produced hydroxyl radicals are able to oxidize major biologically relevant molecules such as proteins, lipids, carbohydrates, and DNA, and are also able to inactivate natural antioxidants (e.g., tocopherol).

**Figure 3:**
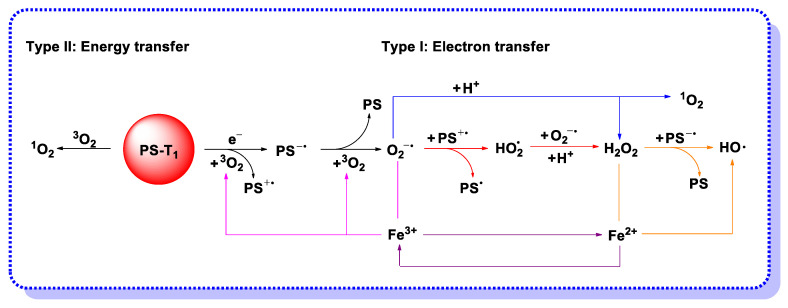
ROS generation mechanism: Type I electron transfer to form free radical ROS and hydrogen peroxide, and Type II energy transfer to form singlet oxygen.

In view of the possible electron transfer from the PS in triplet excited state, the mechanism of hydroxyl radical generation through photocatalysis is proposed and presented in Figure 3. Although H_2_O_2_ is a worse electron acceptor than molecular oxygen, electron transfer reaction from PS* to H_2_O_2_ may occur if the PS triplet state lifetime is long enough [34]. Under the biological conditions with ferrous ions, H_2_O_2_ can undergo the Fenton reaction and produce hydroxyl radicals by one-electron reduction, meanwhile, the produced ferric iron can be reduced back to the ferrous state by superoxide for further use, which is termed the iron-catalyzed Haber–Weiss reaction [35]. On the other hand, H_2_O_2_ can react with the sensitizer radical anion to form hydroxyl radicals and hydroxide anion. The results of the completed and ongoing projects show that PDT is more effective if mechanism I is operative and completed with the generation of highly reactive hydroxyl radicals [36,37,38].

### 2.2. Energy Transfer (Type II) Mechanism

Based on the characteristics of singlet/triplet oxygen electrons and energy levels, the Type II mechanism means the energy transfer from the triplet excited state of an AIE–PS to the ground state of molecular oxygen. Most of the PDT agents produce singlet oxygen (^1^O_2_, ^1^Δ_g_) via a Type II mechanism, which can oxidize the nearby biological species and lead to cytotoxicity. The unoccupied π*_2p_ orbital of these singlet oxygen makes it highly reactive toward electron-rich compounds. For example, ^1^O_2_ can oxidize lipids to produce hydroperoxides, and also can react with amino acids (such as tryptophan, tyrosine, histidine, methionine, cysteine, and cystine) to form hydroperoxides and endoperoxides, which are also the products of oxidation reaction between ^1^O_2_ and deoxyguanosine presented in DNA. The above reactions are subsequently responsible for cellular toxicity, but the cytotoxic effects induced by ^1^O_2_ are independent of the activity of the antioxidant enzymes.

The triplet energies for many AIEgens exceed the energy requirement of the generation of ^1^O_2_ (^1^Δ_g_), which is reported as 22.5 kcal/mol [39,40]. Hence, most AIE–PSs can generate singlet oxygen efficiently [41,42], and the most common mechanism for generating ^1^O_2_ is the quenching process of the PS excited states with molecular oxygen.

## 3. AIE–PSs Based on Electron Transfer (Type I) Mechanism

**Figure 4:**
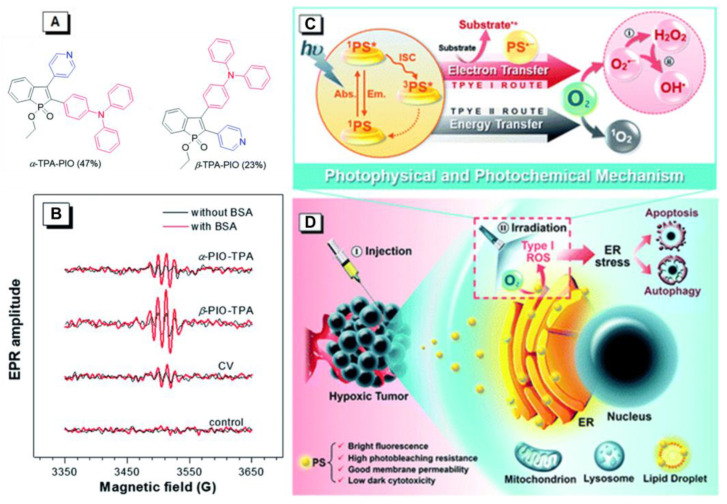
(**A**) Chemical structures of *α*-TPA-PIO and *β*-TPA-PIO. (**B**) Electron paramagnetic resonance (EPR) signals of BMPO (for Type I ROS detection) in the presence of 1 mM *α*-TPA-PIO, *β*-TPA-PIO or CV (without/with 500 nM BSA) in PBS with 1 vol % DMSO. Schematic illustration of (**C**) photophysical and photochemical mechanisms and (**D**) the cytological process of photodynamic therapy (PDT) treatment mediated by phosphindole oxide based fluorogens. (i) Disproportionation reaction; (ii) Haber–Weiss/Fenton reaction. Adapted and modified with permission from reference 44 (open access 2020, Royal Society of Chemistry).

Similar to the development of ACQ–PSs, few reports were available on the capability for Type I ROS generation of AIE–PSs [43]. In 2020, Tang and Zhao reported two new Type I PSs, *α*-TPA-PIO and *β*-TPA-PIO, from phosphindole oxide-based isomers with efficient Type I ROS generation abilities (Figure 4) [44]. The AIE attributes of *α*-TPA-PIO and *β*-TPA-PIO were investigated in DMSO/water mixtures, by employing water as a poor solvent. The emission intensity of *β*-TPA-PIO in pure DMSO solution was very weak. By increasing the fraction of the water, the emission intensity increased gradually and boosted up quickly when the water fraction was over 80%. The emission intensity of the mixture with 99% H_2_O was about 90 times higher than in pure DMSO (λ_em_ = 560 nm). The AIE property of *α*-TPA-PIO was similar to *β*-TPA-PIO. These results indicated that *α*-TPA-PIO and *β*-TPA-PIO were typical AIEgens. To validate the ROS generation ability, electron paramagnetic resonance (EPR) spectroscopy using 5-tert-butoxycarbonyl-5-methyl-1-pyrroline-*N*-oxide (BMPO) as a spin-trap agent was carried out to monitor the formation of oxygenous radicals. EPR showed the typical EPR spectra of oxygenous radical adducts formed with BMPO after white light irradiation of 100 mW cm^−2^ for 5 min, which was in good agreement with those in the literature. *α*-TPA-PIO, *β*-TPA-PIO and crystal violet (CV) exhibited similar BSA-promoted signal enhancements, but *β*-TPA-PIO exhibited the strongest EPR signal intensity, indicative of its best generation ability of oxygenous radicals, namely Type I ROS. These two PSs could be selectively accumulated in a neutral lipid region, particularly in the endoplasmic reticulum (ER) of cells and efficiently induce ER-stress mediated apoptosis and autophagy in PDT. In vivo models indicated that *β*-TPA-PIO successfully achieved remarkable tumor ablation. The ROS-based ER stress triggered by *β*-TPA-PIO-mediated PDT had high potential as a precursor of the immunostimulatory effect for immunotherapy.

## 4. AIE-PSs Based on Energy Transfer (Type II) Mechanism

As the energy transfer process is faster than the electron transfer process, most of the PSs reported so far for AIEgens are singlet oxygen ROS generators. There are several ways to distinguish Type II mechanism from a Type I one. For example, direct monitoring ^1^O_2_ formation involves the detection of its phosphorescence at 1270 nm. Actually, the use of a variety of specific fluorescent probes is the most common way to detect the singlet oxygen generated by AIE–PSs. The following is a summary of the Type II AIE–PSs applied in the design of new ROS generation system for PDT in recent years.

**Figure 5:**
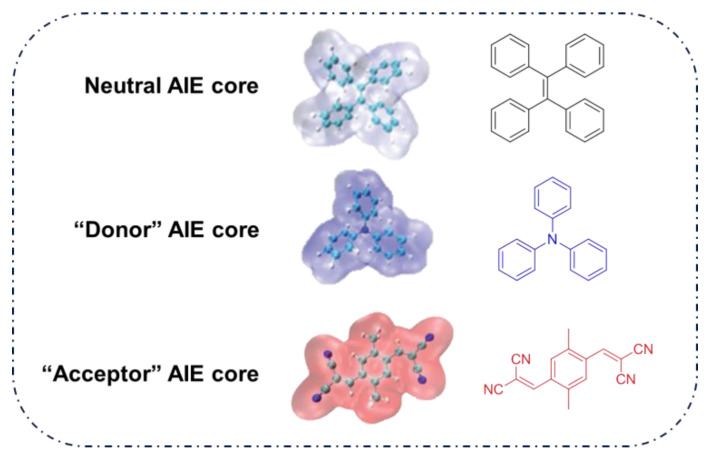
Classification of neutral, donor and acceptor AIE cores by their electron characters, the electronic clouds are represented by colored clouds (the neutral, donor and acceptor AIE cores are represented by the black, blue and red scheme, respectively).

As reported in the literature, the smaller the singlet–triplet energy gap (ΔE_ST_), the higher the ISC rate, and designing molecules with a D-A structure is one of the most effective way to reduce the ΔE_ST_ [45]. In order to illustrate energy transfer in AIE–PSs clearly, the structure of AIE cores are divided into three categories: “neutral” AIE core, “donor” AIE core and “acceptor” AIE core, the latter two are based on the electron withdrawing/donating abilities of the core structures (Figure 5) [46,47,48]. Based on this, AIE–PSs of Type II can be divided into two categories: donor-AIE (neutral)-acceptor and AIE (donor)-acceptor. The photophysical properties, photosensitivity efficiency and singlet oxygen quantum yield of all the AIE–PSs are shown in Table 1.

### 4.1. Donor-AIE (Neutral)-Acceptor PSs

**Figure 6:**
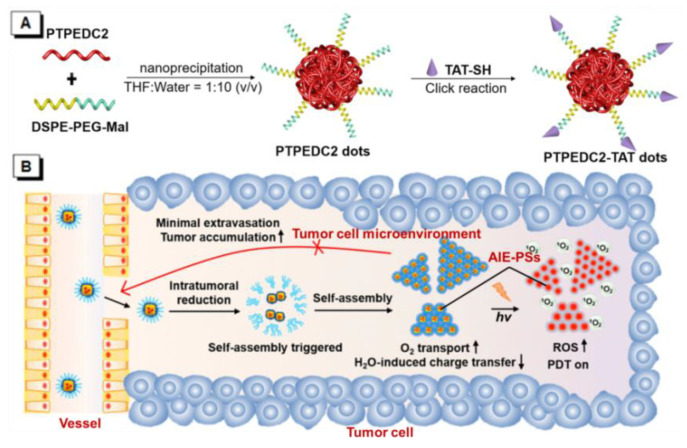
(**A**) Schematic illustration for the synthesis of AIE–PS dots and Aggregation-induced emission trans-activating protein (AIE-TAT) PS dots using PTPEDC2 as an example. Adapted and modified with permission from reference 50 (copyright 2019, American Chemical Society). (**B**) Schematic illustration of in vivo self-assembly of nanoparticle **8**. Adapted and modified with permission from reference 53 (copyright 2020, American Chemical Society).

Table 1 shows the donor-AIE (Neutral)-acceptor PSs that have been reported in recent years [49,50,51,52,53,63]. AIE–PSs **1**–**8** all possess an electron donor and an acceptor on a tetraphenylethene (TPE)-conjugated skeleton, and can induce the effective generation of reactive oxygen species (ROS) for PDT. High ^1^O_2_ generation efficiency can be obtained through inorganic–organic hybridization, chemical linkage, etc. Among them, AIE–PSs **1**–**6** are all constructed in the way of D-π-A, while AIE–PSs **7** and **8** use thiophene and benzothiazole to extend the conjugated structure in the D-π-A structure, which can decrease ΔE_ST_ effectively. The characteristics of several typical PSs of this type are described as follows, for example, NP-1 was prepared via the encapsulate of AIE–PS **1** by encapsulation of AIE–PS **1** using surfactant (DSPE-PEG-MAL) as the polymer matrix, and then followed by the surface decoration of HIV-1 transactivator (RKKRRQRRRC) (Figure 6A) [49]. It could emit near-infrared (NIR) fluorescence centered at 820 nm, and displayed ^1^O_2_ generation ability even greater than that of Ce6, one of the most efficient PSs reported so far [64,65]. Similarly, Liu and co-workers designed and synthesized two conjugated polymer PSs of **4** and **5** based on a small molecule AIE–PS **2** [50]. Subsequently, they were encapsulated by an amphiphilic polymer to yield nanoparticles for both in vitro cancer cell ablation and in vivo zebrafish liver tumor treatment via two-photon excited photodynamic therapy (2PE-PDT) with high efficiency. Under two-photon excitation, **4** and **5** showed high efficiency of ^1^O_2_ production in aqueous solutions and cells due to the enhanced ISC process and molecular conjugation, and among them, **5** presented higher ^1^O_2_ generation efficiency compared with **2**. This two-photon photodynamic therapy could perform precise 3D manipulation of the treatment volume, and provide a target level that cannot be achieved with current treatment technology.

In recent years, many AIE–PS nanoparticles (NPs) have been designed based on the same strategy [52,53]. Moreover, by connecting thiophene ring or benzothiophene ring between the donor and the acceptor structure, AIE–PS with red-shifted emission and better performance can be obtained [66,67]. Wang reported a metal–organic framework (ZIF-8, ZIF = zeolitic imidazolate framework) by AIE–PS **8** assisted in vivo self-assembly nanoplatform, ZIF-8-PMMA-S-S-mPEG (nanoparticle **8**), as an effective tool for organic PS payload to achieve efficient PDT [53]. As shown in Figure 6B, when nanoparticle **8** went through the vessel and entered the tumor tissue owing to the suitable size (about 50 nm), the mPEG chain would cleave under the effect of the disulfide bonds in the tumor, trigger the self-assembly of PS@ZIF-8-PMMA with the AIE-NPs nearby, and promote the retention of AIE–PSs in the tumor significantly because of their relatively large size of 200 nm. When O_2_ was delivered to AIE–PSs, ^1^O_2_ could be produced and maintain in the tumor for a long time. This strategy achieved the largest intratumoral ^1^O_2_ photosensitization of organic PS loaded with organic PS under light activation. It was proved to be advantageous for organic PSs typically suffering from moderate tumor accumulation and compromised intratumoral ROS generation.

### 4.2. AIE (Donor)-Acceptor PSs

Another kind of most widely used core for the preparation of AIE–PSs is triphenylamine and its analogues. Triphenylamine is not only the core of AIE–PSs but also a typical electron donor, so this type of photosensitizers is called “AIE (Donor)-Acceptor PSs”. As reported, triphenylamine moiety has been widely used to enhance the AIE effect of the PSs and pyridinium group is proved to be a specific targeting site toward mitochondria [55,68,69]. The two kinds of moiety are bridged through different units such as phenyl and thiophene groups to regulate the molecular electron effect. As shown in Table 1 (AIE–PSs **9**–**19**), most of the AIE (donor)-acceptor AIE–PSs are constructed in this way and exhibit good performance [42,56,58,59]. For instance, **10** and **12**, respectively, showed strong AIE with the emission enhancement up to 290-fold and 34-fold, large two-photon absorption cross-section up to 477 and 303 GM, respectively, and highly specific targeting to mitochondria in living cells with good biocompatibility [55]. When **10** and **12** were excited at 405 nm, they could emit a light peak at about 800 nm, and produce ROS as well. Therefore, they showed great potential in the clinical application of cell/tissue bioimaging by two-photon fluorescence as well as the image-oriented and mitochondrial photodynamic cancer therapy.

**Figure 7:**
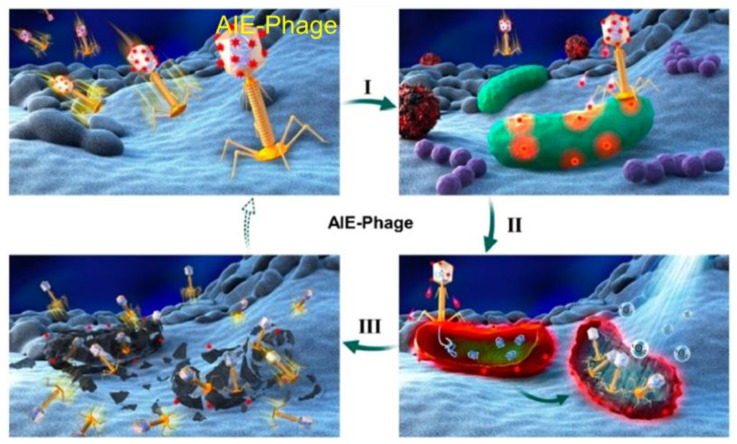
Phage-guided targeting, discriminative imaging, and synergistic killing of bacteria by AIE bioconjugates. The processes are as follows: (**I**) the particular recognition of bacteria by AIEgen conjugated phage; (**II**) the bacterial lighting up imaging by AIE fluorescence and the phage infections as well as the ROS generation of luminogens with aggregation-induced emission (AIEgens); (**III**) the synergistic killing of the target bacteria by phage infections and AIE-based photodynamic inactivation. Adapted and modified with permission from reference 57 (copyright 2020, American Chemical Society).

Besides, Tang et al. proposed a new strategy for imaging and killing certain bacteria at the same time, which was suggested to form a new class of antibacterial conjugated organisms (TVP-PAP) by integrating AIEgens together with bacteriophages (Figure 7). AIE molecule **14** displayed excellent fluorescent characteristics, effective PDI activity and it could be easily modified to the surface of phage entities through a simple amino-carboxyl reaction. The generated TVP-PAP perfectly retained the characteristics of the AIEgen and bacteriophages, and entrusted the inherent AIE fluorescence with both the function of real-time monitoring and bacterial targeting. Moreover, by significantly exceeding two individuals in the bioconjugate, it was endowed with an effective synergistic antibacterial effect [57]. The in vitro test demonstrated that even MDR *P. aeruginosa* could be nearly 100% killed, attributed to the synergistic effect within TVP-PAP.

Moreover, to improve the efficiency of ROS generation, Tang and his co-workers adopted the method of supplying more donor/acceptor electronic cores to construct AIE–PSs [60,61]. As a representative, AIE–PS **20** possessed an ultra-high ^1^O_2_ quantum yield of 98.6% in water; the PS of **20** could efficiently induce cell death in a series of carcinoma cells (IC_50_ values less than 300 × 10^−9^ M) upon irradiation with an extremely low fluence (460 nm, 4 mW cm^−2^ for 10 min) [60]. Atypical structures such as AIE–PS **22** were also classified into this category as they containing -CN groups with electrons that are strongly attractive. Compared with the part that strongly attracts electrons, the other part can be regarded as “donor” core. Photosensitizer **22** showed strong NIR emission centered at 800 nm, good photostability, and high ^1^O_2_ generation efficiency and could induce more tumor cell apoptosis than traditional PSs (CE6) [62]. In addition, Xie et al. rationally designed red-emitting AIE Ir(III) complexes **23** and **24** with different central metal numbers (di-nuclear and tri-nuclear) in 2019. It provided the possibility to develop multi-nuclear Ir(III) complexes with long excitation wavelength for in vivo imaging and PDT [59].

**Figure 8:**
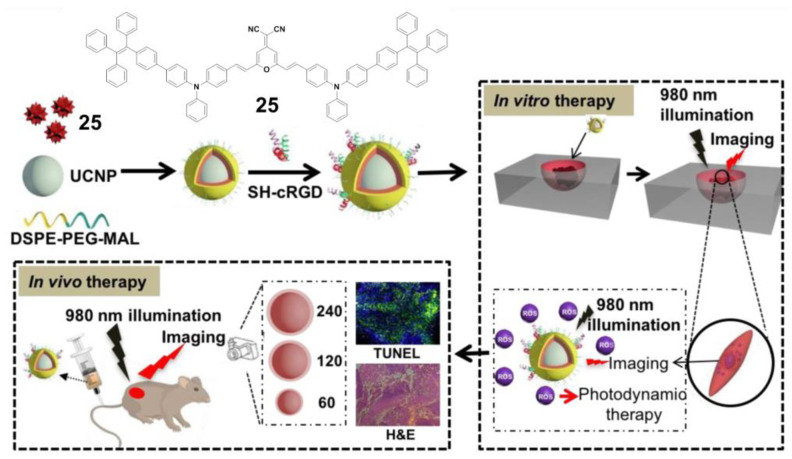
A rational design for a near-infrared (NIR) light-regulated theranostic nanoplatform based on AIE luminogen-encapsulated upconversion nanoparticles. Schematic illustration of preparation of UCNP@**25**-cRGD nanoparticles and their applications in bioimaging and PDT of deep-seated tumors upon NIR laser illumination, in an in vitro three-dimensional (3D) cancer cell spheroid and in a murine tumor model, respectively. Adapted and modified with permission from reference 70 (open access 2019, Theranostics).

Connecting TPE to the atypical AIE (donor)-acceptor structure is also a typical method to construct the near-infrared AIE–PSs. For example, Tang and his team used amphiphilic polymers (i.e., DSPE-PEG2000 and DSPE-PEG2000-MAL) to encapsulate the hydrophobic upconversion nanoparticles (UCNPs) and AIE–PSs **25** (Figure 8) [70]. With high photostability, the prepared UCNP@**25**-cRGD NPs could maintain their fluorescent intensity above 70% after 30 days, and under NIR light illumination, a significant amount of ROS could still be detected even when covered within a 6 mm tissue. The UCNP@**25**-CRGD NPs could specifically target *α*_v_*β*_3_ integrin over-expressed MDA-MB-231 cells and selectively kill these cells in both 2D and 3D cancer models upon NIR light illumination, without obvious dark cytotoxicity. The results of in vivo antitumor evaluation demonstrated that with NIR light illumination, the intravenously injected UCNP@**25**-cRGD NPs could light up the tumors and significantly induce apoptosis of tumor cells, and thus inhibit the growth of large tumors in a mouse model compared to white light illumination.

## 5. Both Energy Transfer and Electron Transfer AIE–PSs

**Figure 9:**
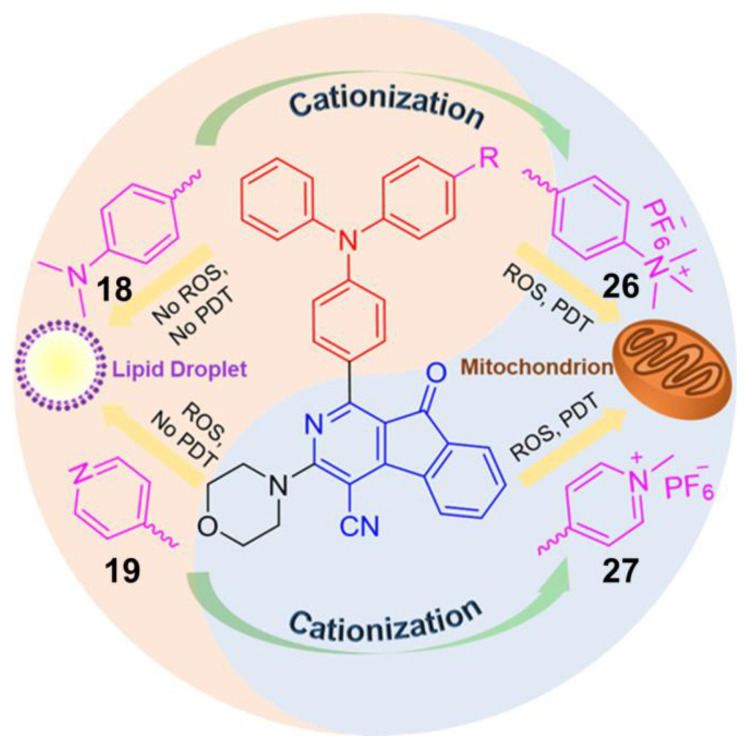
Chemical structures of AIE–PSs **18**, **19, 26,** and **27** (among them, **18** and **19** have low ROS generation efficiency, and **26** and **27** with cations can have high ROS generation efficiency). Adapted and modified with permission from reference 13 (copyright 2019, American Chemical Society).

**Figure 10:**
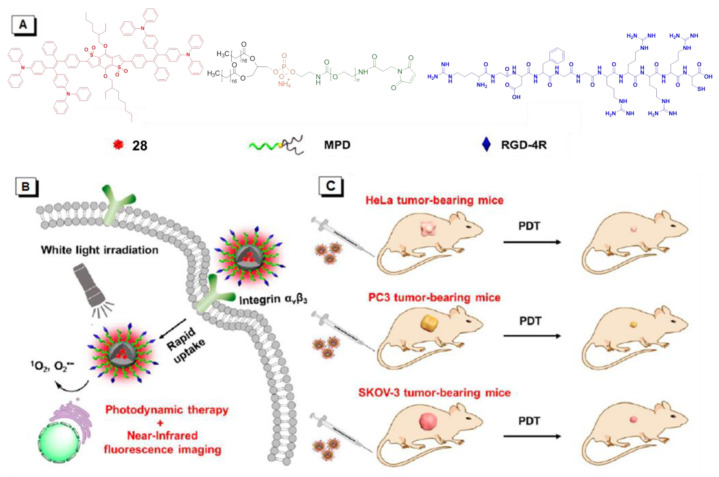
(**A**) Chemical structures of AIE–PS **28**, 1,2-distearoyl-*sn*-glycero-3-phosphoethanolamine -*N*-[maleimide(polyethylene glycol)-2000] (MPD), and RGD-4R. (**B**) Cellular uptake and PDT process of RGD-4R-MPD/**28** NPs. (**C**) Xenograft tumor models of cervical, prostate, and ovarian cancers are established with HeLa, PC3, and SKOV-3 cells, respectively. Adapted and modified with permission from reference 71 (copyright 2020, American Chemical Society).

As mentioned above, ROS mainly includes two types, among which superoxide anion (O_2_^•−^), hydroxyl radical (•OH), peroxide (O_2_^2−^), and so on fall into Type I, while singlet oxygen (^1^O_2_) belongs to Type II. However, there exist some AIE–PSs that can react with oxygen to form both O_2_^•−^/•OH/O_2_^2−^ and ^1^O_2_. For example, AIE –PSs **18** and **19** mentioned in Section 4.2 are mainly PSs of Type II, but their protonated products, **26** and **27** NPs are quite different (Figure 9) [13]. Through research, it is found that the ROS and ^1^O_2_ generation capacity of **19** and **27** NPs are inconsistent. Tang suggested that **27** produces not only ^1^O_2_ but also ROS. To explain this observation, sodium azide (NaN_3_) as a ^1^O_2_ quencher was added to the 9, 10-anthracenediylbis (methylene)dimalonic acid (ABDA) solution in the presence of **19**, **26**, or **27** NPs. Gradually, the absorption of ABDA with **26**, or **27** declined with prolonged irradiation time even at a high concentration of NaN_3_ (240 mg/mL). In addition, dihydroethidium (DHE) was used as a sensitive O_2_^•−^ probe to assess the O_2_^•−^ generation in HeLa cells by flow cytometry. Results showed that the fluorescence of HeLa cells with DHE became stronger in the presence of **26**, or **27** under light irradiation. Clearly, **26** and **27** could generate a small amount of O_2_^•−^, which is the precursor of •OH and H_2_O_2_. Tang also used the indicator to detect the type of active oxygen. AIE–PS **28** exhibited a prominent AIE property with strong NIR fluorescence in aggregates and was capable of efficiently generating ROS of O_2_^•−^ and ^1^O_2_ under white light irradiation (Figure 10) [71]. To demonstrate the AIE property of **28**, its emission behavior in THF/water mixtures with different volume fractions of water (*f_w_*, vol %) was examined. The results showed that the emission peak of **28** moved to 690 nm and intensified greatly in the mixture with *f_w_* of 90, indicating a prominent AIE characteristic. This type of NIR photosensitizer with AIE characteristic could be a promising alternative for the fabrication of nanoprobes and nanomedicines for the potential clinical applications of multiple tumors.

**Figure 11:**
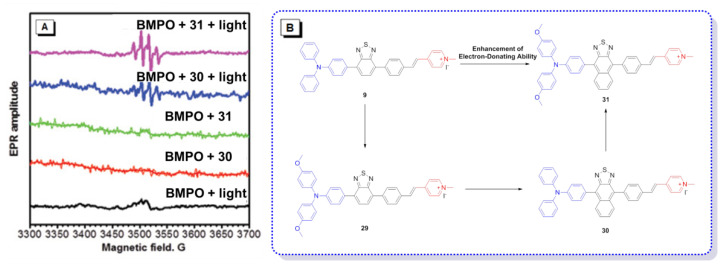
(**A**) EPR signals of BMPO for free radical ROS characterization. Adapted and modified with permission from reference 54 (copyright 2020, Wiley-VCH). (**B**) Chemical structures of AIE–PSs **9**, **29**–**31** (the donor and acceptor groups are represented by the blue and red scheme, respectively).

In addition to the usage of indicators to detect the type of ROS, the operation of instruments is also completely feasible. As shown in Figure 11A, EPR spectroscopy was used to provide solid evidence for the free radical ROS generation of four AIE–PSs [54]. Both BMPO under irradiation and BMPO + AIEgens (**30** and **31**) in the dark did not produce any signal in the EPR spectra. In contrast, significant EPR signals could be observed upon irradiation of the BMPO + **30**/**31** solution, associated with the production of paramagnetic free radical species. Thus, it could be confirmed that **9** with low intramolecular charge transfer (ICT) produced only ^1^O_2_, species. According to the results derived from **29** and naphtho[2,3-c][1,2,5]thiadiazole-based (NZ-based) AIE–PSs, once the ICT intensity was enhanced by a donor with strengthened electron-releasing ability (Figure 11B), the ^1^O_2_, species were transformed into free radical ROS, which agreed well with the proposal of “more ICT leading to the generation of more free radical ROS”.

## 6. Conclusions and Outlooks

As a noninvasive treatment, PDT has been extensively studied for both cancer diagnosis and therapy. Notably, the therapeutic effect of PDT is directly influenced by the ROS-generation ability of PSs. The emerging AIE–PSs and their application for image-guided therapy have shown evident advantages over the classical ones and thus have attracted much attention recently. In recent reports, the classification of the Type I and Type II AIE–PSs oxidation reactions is still not clearly categorized. Thus, in this brief review, we have tried to classify the AIE–PSs according to the following standards: (1) Type I: AIE–PSs generates superoxide anion (O_2_^•−^), hydroxyl radical (•OH), peroxide (O_2_^2−^), and other free radical ROS through electron transfer. Electron transfer can actually occur in either direction, but most commonly, the excited sensitizer acts as an oxidant. Therefore, this type of AIE–PSs can achieve the purpose of PDT under hypoxic conditions. (2) Type II: AIE–PSs generate singlet oxygen (^1^O_2_) through energy transfer. This process must be under the condition of concentrated oxygen to convert triplet oxygen into singlet oxygen. (3) Type I and Type II: AIE–PSs generate both free radical ROS and ^1^O_2_ through electron transfer and energy transfer, respectively. In the case of Type II, electron transfer occurs from the AIE–PSs to oxygen, and the generation of oxidation sensitizer and O_2_^•−^ is also assigned to this category. Simultaneously, we classify the Type II PSs into two categories: donor-AIE (neutral)-acceptor and AIE (donor)-acceptor. The construction principle is to form a D-A and/or a D-π-A structure.

The reported that AIE–PSs have rendered new power to PDT, however, it could be noticed that most of the AIE–PSs used in PDT nowadays are Type II PSs. In fact, the development of Type II PSs will be seriously influenced by the fundamental paradox which existed between the high O_2_ dependency of AIE–PSs and the hypoxic (not anaerobic) nature around solid tumors caused by insufficient blood supply. Furthermore, during the PDT process, especially in the continuous treatment, the photochemical consumption of O_2_ as well as the micro-vascular damage will aggravate the O_2_ shortage, and restrict the effect of PDT to an unsatisfactory outcome. An effective pathway to solve this problem is to design new PSs with low requirement of O_2_ and can adapt to the hypoxic environment around the tumor tissues. Compared to the Type II PSs, Type I PSs with stronger hypoxia tolerance are a good choice, so the subsequent development of Type I AIE–PSs is full of opportunities and challenges in the future. In addition, methods for distinguishing two different mechanism types of ROS generation are also imperative to provide an insight into the ROS formation mechanism of AIE–PSs. If only considering the effect of medical treatment, the development of two-photon AIE–PSs with high reactive oxygen generation efficiency is also crucial.

## Figures and Tables

**Table 1:** Chemical structures, absorption/emission peaks, ΔE_ST_, consuming rate of ABDA, treatment efficiency and singlet oxygen quantum yield of different AIE–PSs (the donor and acceptor groups of different AIE–PSs are represented by the blue and red scheme, respectively).

AIE-PSs	Chemical Structures	Absorption/Emission [nm]	ΔE_ST_ [eV] ^(a)^	Consuming Rate of ABDA	Treatment Efficiency	^1^O_2_ Quantum Yield ^(b)^	Ref.
**Donor-AIE (Neutral)-Acceptor AIE–PSs**							
1	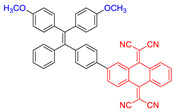	520/820	0.067	3.58 nmol min^−1^	IC_50_ = 5.0 μg mL^−1^ (4T1 cell) White light: 60 mW cm^−2^ for 5 min	0.08	[49]
2	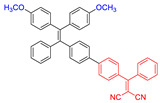	410/620	0.23	3.98 nmol min^−1^	IC_50_ = 8.0 μg mL^−1^ (4T1 cell) White light: 60 mW cm^−2^ for 5 min	-	[50]
3	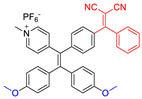	440/606	-	-	IC_50_ = 1.5 × 10^−6^ M (HeLa cell) White light: 36 mW cm^−2^ for 40 min	-	[51]
4	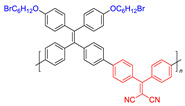	410/610	-	9.03 nmol min^−1^	-	-	[50]
5	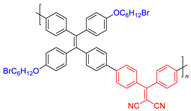	410/620	-	21.8 nmol min^−1^	-	0.55	[50]
6	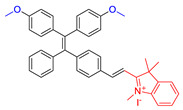	480/710	0.63	-	IC_50_ = 70.72 μg mL^−1^ polymer (HeLa cell) White light: 100 mW cm^−2^ for 5 min	0.89	[52]
7	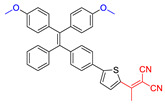	450/651	0.50	12.0 nmol min^−1^	-	-	[45]
8	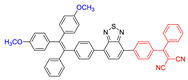	450/650	0.33	27.0 nmol min^−1^	IC_50_ = 1 μg mL^−1^ (MDA-MB-231) White light: 60 mW cm^−2^ for 5 min	Higher than 0.08	[53]
**AIE (Donor)-Acceptor AIE–PSs**							
9	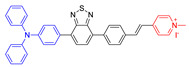	447/625	0.15	-	-	-	[54]
10	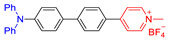	442/558	-	70.20 nmol min^−1^	55% killing effect of HeLa cell 10 × 10^−6^ M White light: 60 mW cm^−2^ for 30 min	3.17	[55]
11	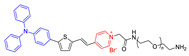	480/680	-	-	IC_50_ = 10.0 μg mL^−1^ (4T1 cell) White light: 24 mW cm^−2^ for 30 min	-	[56]
12	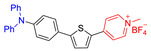	448/592	-	121.74 nmol min^−1^	75% killing effect of HeLa cell 10 × 10^−6^ M White light: 60 mW cm^−2^ for 30 min	3.71	[55]
13	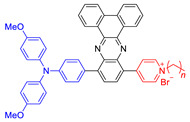	508/720	0.093	-	83.2% killing effect of *E. Coli* 10 × 10^−6^ M White light: 60 mW cm^−2^ for 10 min	0.71	[42]
14	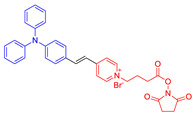	457/630	-	-	79% killing effect of *P. aeruginosa* 10 × 10^−6^ M White light: 4.2 mW cm^−2^ for 30 min	0.72	[57]
15	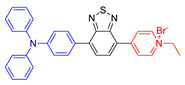	486/705	0.79	18.2 nmol min^−1^	98.4% killing effect of *E. Coli* 10 × 10^−6^ M White light: 4.2 mW cm^−2^ for 30 min	-	[58]
16	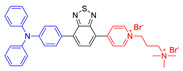	493/685	-	15.3 nmol min^−1^	87.3% killing effect of *E. Coli* 10 × 10^−6^ M White light: 4.2 mW cm^−2^ for 30 min	-	[58]
17	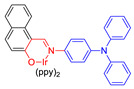	469/652	-	-	IC_50_ = 1.1 × 10^−^^5^ M (HeLa cell) White light: 20 mW cm^−2^ for 30 min	-	[59]
18	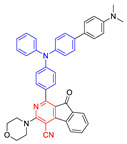	417/601	0.09	-	-	-	[13]
19	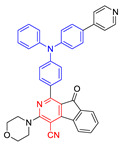	409/593	0.16	0.7 nmol min^−1^	-	-	[13]
20	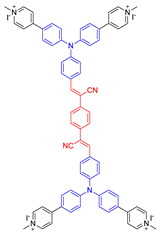	405/580	-	-	IC_50_ = 3.0 × 10^−^^7^ M (HeLa cell) White light: 4 mW cm^−2^ for 10 min	0.98	[60]
21	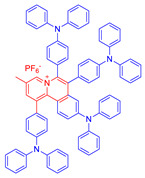	440/590	0.55	-	More than 70% killing effect of *E. Coli* 5 × 10^−6^ M White light: 10 mW cm^−2^ for 15 min	0.98	[61]
22	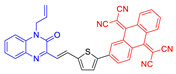	530/800	0.12	-	IC_50_ = 3.26 μg mL^−1^ (Hela cell) White light: 100 mW cm^−2^ for 5 min	0.63	[62]
23	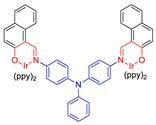	469/671	-	-	IC_50_ = 6.3 × 10^−^^6^ M (HeLa cell) White light: 20 mW cm^−2^ for 30 min	-	[59]
24	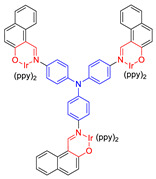	469/690	-	-	IC_50_ = 1.1 × 10^−^^6^ M (HeLa cell) White light: 20 mW cm^−2^ for 30 min	-	[59]

^(a)^ The data of singlet-triplet energy gap were theoretical calculation results according to references; ^(b^^)^ quantum yield was estimated in reference to Rose Bengal.

## Data Availability

Data available in a publicly accessible repository.
